# Supplementary material for: Modeling glioblastoma heterogeneity as a dynamic network of cell states
Source: Mol Syst Biol. 2021 Sep 16;17(9):e10105. doi: 10.15252/msb.202010105 (PMC8444284; doi:10.15252/msb.202010105)
Supplement: Supplementary file 6 — Source Data for Figure 5 [file MSB-17-e10105-s004.zip › Figure5A_sourcedata/GSEA_3071/nosigdb_stateV.GseaPreranked.1622795159954/IVY_CT.html]

Details for gene set IVY\_CT[GSEA]

|  || Dataset | state43071 |
| Phenotype | NoPhenotypeAvailable |
| Upregulated in class | na\_pos |
| GeneSet | IVY\_CT |
| Enrichment Score (ES) | 0.7180886 |
| Normalized Enrichment Score (NES) | 4.0926313 |
| Nominal p-value | 0.0 |
| FDR q-value | 0.0 |
| FWER p-Value | 0.0 |
Table: GSEA Results Summary

  

Fig 1: Enrichment plot: IVY\_CT      
 Profile of the Running ES Score & Positions of GeneSet Members on the Rank Ordered List

  

| PROBE | GENE SYMBOL | GENE\_TITLE | RANK IN GENE LIST | RANK METRIC SCORE | RUNNING ES | CORE ENRICHMENT || 1 | HIST1H1B |  |  | 0 | 2.500 | 0.0171 | Yes |
| 2 | ESCO2 |  |  | 3 | 2.193 | 0.0316 | Yes |
| 3 | FAM111B |  |  | 4 | 2.012 | 0.0453 | Yes |
| 4 | HMGB2 |  |  | 5 | 2.011 | 0.0591 | Yes |
| 5 | NUSAP1 |  |  | 6 | 2.001 | 0.0727 | Yes |
| 6 | IGFBP2 |  |  | 8 | 1.922 | 0.0856 | Yes |
| 7 | POU3F2 |  |  | 11 | 1.891 | 0.0981 | Yes |
| 8 | PTPRZ1 |  |  | 13 | 1.865 | 0.1107 | Yes |
| 9 | TOP2A |  |  | 14 | 1.842 | 0.1232 | Yes |
| 10 | DHFR |  |  | 18 | 1.795 | 0.1349 | Yes |
| 11 | HIST1H1D |  |  | 19 | 1.786 | 0.1471 | Yes |
| 12 | NCAPG |  |  | 21 | 1.760 | 0.1589 | Yes |
| 13 | RAD51AP1 |  |  | 22 | 1.757 | 0.1709 | Yes |
| 14 | CKB |  |  | 23 | 1.748 | 0.1828 | Yes |
| 15 | CENPF |  |  | 28 | 1.705 | 0.1936 | Yes |
| 16 | CCNE2 |  |  | 32 | 1.664 | 0.2044 | Yes |
| 17 | RRM2 |  |  | 34 | 1.660 | 0.2155 | Yes |
| 18 | MYBL2 |  |  | 35 | 1.651 | 0.2268 | Yes |
| 19 | ASCL1 |  |  | 43 | 1.604 | 0.2364 | Yes |
| 20 | KIF15 |  |  | 52 | 1.568 | 0.2455 | Yes |
| 21 | PBK |  |  | 54 | 1.536 | 0.2558 | Yes |
| 22 | EZH2 |  |  | 55 | 1.534 | 0.2663 | Yes |
| 23 | GINS2 |  |  | 57 | 1.528 | 0.2765 | Yes |
| 24 | DTL |  |  | 60 | 1.503 | 0.2863 | Yes |
| 25 | GTSE1 |  |  | 66 | 1.491 | 0.2955 | Yes |
| 26 | COL9A3 |  |  | 68 | 1.488 | 0.3055 | Yes |
| 27 | CDK1 |  |  | 69 | 1.488 | 0.3156 | Yes |
| 28 | PTN |  |  | 70 | 1.482 | 0.3258 | Yes |
| 29 | FREM2 |  |  | 74 | 1.453 | 0.3351 | Yes |
| 30 | HIST1H2AH |  |  | 80 | 1.424 | 0.3438 | Yes |
| 31 | FBXO5 |  |  | 82 | 1.422 | 0.3533 | Yes |
| 32 | CLSPN |  |  | 84 | 1.417 | 0.3628 | Yes |
| 33 | SCRG1 |  |  | 87 | 1.404 | 0.3720 | Yes |
| 34 | HES6 |  |  | 91 | 1.391 | 0.3809 | Yes |
| 35 | MCM10 |  |  | 92 | 1.389 | 0.3904 | Yes |
| 36 | CDCA2 |  |  | 96 | 1.382 | 0.3992 | Yes |
| 37 | CENPM |  |  | 114 | 1.337 | 0.4050 | Yes |
| 38 | TACC3 |  |  | 116 | 1.334 | 0.4139 | Yes |
| 39 | WSCD1 |  |  | 126 | 1.322 | 0.4212 | Yes |
| 40 | SMC4 |  |  | 127 | 1.320 | 0.4302 | Yes |
| 41 | LRRN3 |  |  | 133 | 1.307 | 0.4382 | Yes |
| 42 | SOX2 |  |  | 147 | 1.279 | 0.4444 | Yes |
| 43 | GAS1 |  |  | 154 | 1.263 | 0.4518 | Yes |
| 44 | CENPK |  |  | 162 | 1.241 | 0.4589 | Yes |
| 45 | LMNB1 |  |  | 165 | 1.238 | 0.4670 | Yes |
| 46 | DNMT1 |  |  | 166 | 1.237 | 0.4754 | Yes |
| 47 | CDCA3 |  |  | 169 | 1.230 | 0.4834 | Yes |
| 48 | ATP6V0E2 |  |  | 181 | 1.209 | 0.4895 | Yes |
| 49 | KIF4A |  |  | 191 | 1.193 | 0.4959 | Yes |
| 50 | NUF2 |  |  | 192 | 1.189 | 0.5040 | Yes |
| 51 | FANCI |  |  | 196 | 1.179 | 0.5115 | Yes |
| 52 | ASPM |  |  | 207 | 1.163 | 0.5175 | Yes |
| 53 | PRC1 |  |  | 211 | 1.161 | 0.5248 | Yes |
| 54 | UBE2C |  |  | 214 | 1.154 | 0.5323 | Yes |
| 55 | CDKN2C |  |  | 223 | 1.143 | 0.5385 | Yes |
| 56 | SOX8 |  |  | 231 | 1.136 | 0.5449 | Yes |
| 57 | KLHDC8A |  |  | 234 | 1.134 | 0.5523 | Yes |
| 58 | ASF1B |  |  | 237 | 1.130 | 0.5596 | Yes |
| 59 | NDC80 |  |  | 240 | 1.129 | 0.5669 | Yes |
| 60 | TPX2 |  |  | 248 | 1.122 | 0.5732 | Yes |
| 61 | KCNQ2 |  |  | 270 | 1.091 | 0.5765 | Yes |
| 62 | KIF14 |  |  | 272 | 1.090 | 0.5838 | Yes |
| 63 | BUB1 |  |  | 277 | 1.084 | 0.5904 | Yes |
| 64 | CKAP2L |  |  | 285 | 1.078 | 0.5964 | Yes |
| 65 | NLGN1 |  |  | 294 | 1.072 | 0.6021 | Yes |
| 66 | DLL3 |  |  | 315 | 1.048 | 0.6054 | Yes |
| 67 | CDCA7 |  |  | 330 | 1.038 | 0.6098 | Yes |
| 68 | TTK |  |  | 383 | 0.966 | 0.6063 | Yes |
| 69 | UHRF1 |  |  | 390 | 0.958 | 0.6117 | Yes |
| 70 | SOX21 |  |  | 392 | 0.957 | 0.6180 | Yes |
| 71 | NOVA1 |  |  | 393 | 0.957 | 0.6245 | Yes |
| 72 | SPATA6 |  |  | 404 | 0.946 | 0.6290 | Yes |
| 73 | LRRN1 |  |  | 405 | 0.946 | 0.6355 | Yes |
| 74 | DLGAP5 |  |  | 418 | 0.936 | 0.6395 | Yes |
| 75 | OLIG1 |  |  | 430 | 0.924 | 0.6437 | Yes |
| 76 | CENPE |  |  | 437 | 0.918 | 0.6488 | Yes |
| 77 | METRN |  |  | 450 | 0.907 | 0.6527 | Yes |
| 78 | SCD5 |  |  | 485 | 0.879 | 0.6521 | Yes |
| 79 | BLM |  |  | 486 | 0.879 | 0.6580 | Yes |
| 80 | TCF12 |  |  | 512 | 0.855 | 0.6590 | Yes |
| 81 | H19 |  |  | 530 | 0.848 | 0.6615 | Yes |
| 82 | BUB1B |  |  | 535 | 0.846 | 0.6665 | Yes |
| 83 | NPAS3 |  |  | 539 | 0.842 | 0.6717 | Yes |
| 84 | NCAN |  |  | 540 | 0.842 | 0.6774 | Yes |
| 85 | BIRC5 |  |  | 541 | 0.842 | 0.6832 | Yes |
| 86 | SALL1 |  |  | 545 | 0.836 | 0.6883 | Yes |
| 87 | ITGB8 |  |  | 564 | 0.822 | 0.6904 | Yes |
| 88 | ID4 |  |  | 568 | 0.819 | 0.6954 | Yes |
| 89 | HIST1H2AG |  |  | 569 | 0.818 | 0.7010 | Yes |
| 90 | FAM181B |  |  | 600 | 0.800 | 0.7006 | Yes |
| 91 | TTYH1 |  |  | 611 | 0.794 | 0.7041 | Yes |
| 92 | PCDHGC3 |  |  | 614 | 0.791 | 0.7091 | Yes |
| 93 | OIP5 |  |  | 620 | 0.788 | 0.7135 | Yes |
| 94 | C1orf61 |  |  | 625 | 0.787 | 0.7181 | Yes |
| 95 | DSEL |  |  | 683 | 0.756 | 0.7122 | No |
| 96 | SOX11 |  |  | 690 | 0.751 | 0.7161 | No |
| 97 | AURKA |  |  | 761 | 0.718 | 0.7075 | No |
| 98 | RNASEH2A |  |  | 859 | 0.674 | 0.6932 | No |
| 99 | KCNIP1 |  |  | 874 | 0.669 | 0.6951 | No |
| 100 | SOX9 |  |  | 905 | 0.658 | 0.6938 | No |
| 101 | EDNRB |  |  | 911 | 0.654 | 0.6972 | No |
| 102 | MELK |  |  | 915 | 0.653 | 0.7011 | No |
| 103 | PTTG1 |  |  | 936 | 0.647 | 0.7016 | No |
| 104 | DCLK2 |  |  | 957 | 0.638 | 0.7021 | No |
| 105 | NRM |  |  | 1031 | 0.613 | 0.6921 | No |
| 106 | CDCA8 |  |  | 1043 | 0.610 | 0.6942 | No |
| 107 | DDR1 |  |  | 1064 | 0.603 | 0.6944 | No |
| 108 | BCHE |  |  | 1116 | 0.588 | 0.6885 | No |
| 109 | DBI |  |  | 1118 | 0.588 | 0.6923 | No |
| 110 | SHOX2 |  |  | 1128 | 0.586 | 0.6946 | No |
| 111 | PCBP4 |  |  | 1163 | 0.574 | 0.6919 | No |
| 112 | GATM |  |  | 1192 | 0.565 | 0.6903 | No |
| 113 | HIST1H2AM |  |  | 1196 | 0.563 | 0.6936 | No |
| 114 | OLIG2 |  |  | 1213 | 0.559 | 0.6943 | No |
| 115 | CSPG5 |  |  | 1263 | 0.541 | 0.6885 | No |
| 116 | KIF2C |  |  | 1309 | 0.530 | 0.6834 | No |
| 117 | NES |  |  | 1325 | 0.527 | 0.6840 | No |
| 118 | CCNB2 |  |  | 1373 | 0.513 | 0.6784 | No |
| 119 | SEMA6A |  |  | 1375 | 0.512 | 0.6817 | No |
| 120 | SAPCD2 |  |  | 1455 | 0.491 | 0.6697 | No |
| 121 | MSI1 |  |  | 1601 | 0.459 | 0.6448 | No |
| 122 | METTL7B |  |  | 1609 | 0.458 | 0.6465 | No |
| 123 | MEX3A |  |  | 1667 | 0.443 | 0.6385 | No |
| 124 | CRISPLD1 |  |  | 1697 | 0.437 | 0.6358 | No |
| 125 | RFX4 |  |  | 1773 | 0.424 | 0.6242 | No |
| 126 | NLGN3 |  |  | 2078 | 0.376 | 0.5678 | No |
| 127 | FADS2 |  |  | 2155 | 0.367 | 0.5555 | No |
| 128 | ABAT |  |  | 2170 | 0.365 | 0.5553 | No |
| 129 | MLC1 |  |  | 2197 | 0.362 | 0.5527 | No |
| 130 | SRPX |  |  | 2646 | 0.305 | 0.4679 | No |
| 131 | AQP4 |  |  | 2956 | 0.273 | 0.4098 | No |
| 132 | ETV1 |  |  | 2979 | 0.271 | 0.4074 | No |
| 133 | IGF2BP3 |  |  | 2983 | 0.271 | 0.4087 | No |
| 134 | SLC10A4 |  |  | 3015 | 0.267 | 0.4045 | No |
| 135 | GRIK3 |  |  | 3138 | 0.256 | 0.3826 | No |
| 136 | S100A3 |  |  | 3664 | -0.325 | 0.2829 | No |
| 137 | LFNG |  |  | 3749 | -0.339 | 0.2690 | No |
| 138 | VCAN |  |  | 4491 | -0.537 | 0.1289 | No |
| 139 | FERMT1 |  |  | 4549 | -0.564 | 0.1217 | No |
| 140 | MATN2 |  |  | 5200 | -1.557 | 0.0062 | No |
| 141 | EGFR |  |  | 5230 | -1.765 | 0.0126 | No |
Table: GSEA details [plain text format]

  

Fig 2: IVY\_CT: Random ES distribution      
 Gene set null distribution of ES for **IVY\_CT**
